# Studies on the Structure and Properties of Ultrasound-Assisted Enzymatic Digestion of Collagen Peptides Derived from *Chinemys reevesii* Skin

**DOI:** 10.3390/foods14172960

**Published:** 2025-08-25

**Authors:** Wenzhuo Chen, Dandan Yu, Li Guan, Hui Cao

**Affiliations:** College of Tourism and Culinary Science, Yangzhou University, Yangzhou 225127, China; mx120231295@stu.yzu.edu.cn (W.C.); yudandan981004@163.com (D.Y.); gl124101@163.com (L.G.)

**Keywords:** ultrasound, collagen, hydrolysates, peptides, structure, antioxidant

## Abstract

This study examined the ultrasound pretreatment (UP) and simultaneous ultrasound (US) effects on the structural–functional features of collagen peptides in *Chinemys reevesii* skin collagen hydrolysates (CCHs) using a composite protease system (Trypsin: Alkaline protease, 1:1). Structural characterization revealed that UP induced the unfolding of collagen molecules, evidenced by reduced disulfide bond content and the concomitant increase in surface hydrophobicity. Consequently, ultrasound pretreatment-assisted enzymatic hydrolysis (UPH) significantly enhanced the yield of low-molecular-weight components (<0.18 kDa) and hydrophobic amino acids, which rose by 3.03% and 4.89% compared to the results of conventional enzymatic treatment (CE). UPH showed higher antioxidant activity than CE and WUH over CE and whole-process ultrasound-assisted hydrolysates (WUH). At 5 mg/mL, it displayed an ABTS radical scavenging rate of 87.59%, a DPPH scavenging rate of 53.37%, and the highest reducing power. However, WUH induced peptide reaggregation due to prolonged ultrasonication, thus exhibiting moderately lower antioxidant activity than UPH. These findings suggest that UP is an effective strategy to optimize the structure and composition of CCHs, outperforming both CE and WUH in facilitating the release of antioxidant peptides and improving antioxidant capacity.

## 1. Introduction

The majority of collagen found in nature is sourced from the bones and skin of terrestrial mammals and aquatic species including, but not limited to, pigs, cattle, sheep, fish, and squid [1,2]. Nevertheless, incidents involving diseases such as bovine spongiform encephalopathy (BSE), transmissible spongiform encephalopathy (TSE), foot-and-mouth disease (FMD), and avian influenza (AI) have seriously eroded public trust in the safety profile of collagen sourced from terrestrial animals [3]. However, few studies have been conducted on collagen sourced from diseased aquatic animals, particularly regarding disease-related safety implications, suggesting they may represent a more secure alternative. Aquatic collagen has garnered increasing scientific and industrial interest owing to its high bioavailability, biocompatibility, and wide availability, as well as the absence of religious restrictions. This growing attention has spurred extensive research efforts to characterize collagen from various marine sources and evaluate its functional properties. Aquatic organisms, including fish, jellyfish, sea cucumbers, starfish, mantis shrimp, sponges, squid, and their processing by-products, offer a sustainable pathway for sourcing marine collagen [4,5,6,7,8,9,10]. Unlike cold-water fish, turtles are ectothermic amniotic reptiles adapted to warmer aquatic environments [11]. Their collagen exhibits a higher denaturation temperature, conferring greater thermal stability compared to other aquatic collagens across diverse applications [12]. Global turtle production reached 355,000 tons in 2014 [13], with *Chinemys reevesii* representing a commercially significant species. Native to China, Japan, and Korea, this species possesses substantial nutritional and medicinal value [13]. Notably, artificially bred *Chinemys reevesii* are explicitly excluded from endangered species protection categories, as their large-scale commercial farming in China has ensured sustainable populations. This legal and ecological status designates them as legitimate, high-quality raw materials for tortoise shell glue production, distinguishing them from wild protected species. Tortoise shell glue is renowned in traditional medicine as a hematinic and hemostatic agent, historically being employed as a vital tonic [14]. During its production, turtle skin emerges as a major byproduct, representing a rich yet underutilized source of collagen. Enzymatic extraction studies have demonstrated that turtle skin yields 51.68% collagen, with glycine constituting the predominant amino acid [15]. The direct disposal of tortoise skin poses a risk of environmental contamination and leads to the substantial wastage of biological resources. Comparative analyses reveal that turtle skin collagen contains higher hydroxyproline content and demonstrates a superior nutritional profile relative to porcine collagen. Concurrently, this collagen source also readily forms bioaccessible small peptides, which exhibit enhanced intestinal absorption and bioavailability [16].

Collagen peptides have demonstrated substantial improvements in digestibility, absorbability, nutritional value, and functional properties compared to native collagen [17]. Zhang [18] reported that 66.23% of peptides in the pepsin hydrolysate of Chinese soft-shelled turtle calipash collagen had molecular weights below 2 kDa. This molecular profile correlates strongly with bioavailability, as demonstrated by Wu et al. [19], who reported 88.8% intestinal absorption efficiency for collagen peptides under 2 kDa. Collagen peptides are widely recognized for their antioxidant, anti-fatigue, hypoglycemic, hypotensive, antibacterial, and other bioactive functions [20]. For instance, Wang et al. [21] discovered that forest frog skin collagen peptides possess bacteriostatic properties. Wang et al. [22] purified six antioxidant peptides from redlip croaker scale collagen digests, among which GPEGPMGLE, EGPFGPEG and GFIGPTE exhibited strong antioxidant properties. These findings have spurred the growth of commercial applications, with collagen peptides being increasingly incorporated as functional ingredients into nutritional supplements, fortified foods and beverages, as well as into cosmetic, nutraceutical, and pharmaceutical formulations [3,23,24]. Additionally, aquatic collagen is characterized by a less robust structure and lower thermal stability when juxtaposed with mammalian collagen [12,25]. These structural attributes render it more susceptible to proteolytic hydrolysis, thereby facilitating the generation of bioactive peptides [26]. Islam et al. [27] reported that papain-hydrolyzed peptides from *Chinemys reevesii* demonstrated higher DPPH scavenging activity than pork and greater reducing power than engraved catfish.

Ultrasound represents a promising, eco-friendly technique that triggers conformational alterations in secondary or tertiary protein structures via a cavitation mechanism. This mechanism expands the protein framework and unveils additional active sites, thereby enhancing enzymatic hydrolysis efficiency and the bioactivity of resulting hydrolysates. Indriani et al. [28] found that ultrasound pretreatment and simultaneous treatment-assisted enzymatic digestion enhanced the production efficiency of collagen peptides from bullfrog skin and imparted enhanced antioxidant activity. Similarly, moderate ultrasound-assisted preparation of *Micrococcus* sp. antioxidative peptides exhibited superior free radical scavenging activity compared to conventional enzymatic hydrolysis, with significantly enhanced rates against both DPPH and OH radicals [29]. Differently, in this study, a trypsin and alkaline protease complex enzymatic system was added to different ultrasound modes to promote the release of more bioactive peptides. There are few previous literature reports on collagen peptides from turtle skin.

Therefore, this study aims to characterize the structural modifications induced by ultrasound-assisted complex enzymatic hydrolysis in collagen peptides derived from *Chinemys reevesii* skin and assess their potential to enhance antioxidant activity. 
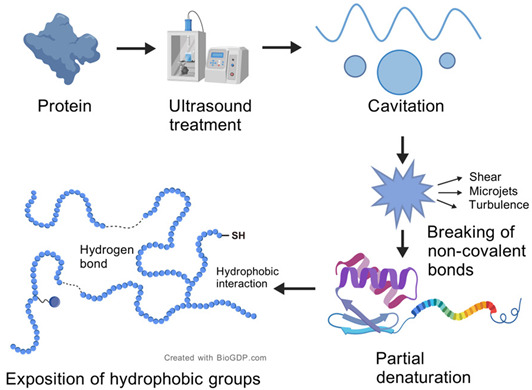
 Effects of ultrasound on protein structure (created with BioGDP.com [30]).

## 2. Materials and Methods

### 2.1. Materials and Chemicals

*Chinemys reevesii* samples were purchased from Maoda Ecological Aquaculture Co., Ltd. (Yangzhou, China). Acetonitrile of a chromatographic grade was purchased from Shanghai Aladdin Biochemical Technology Co., Ltd. (Shanghai, China). Trypsin (250 U/mg) and alkaline protease (200 U/mg) were purchased from Beijing Solebo Technology Co., Ltd. (Beijing, China). All other chemicals used in this study were of an analytical grade.

### 2.2. Methods

#### 2.2.1. Preparation of Turtle Skin Collagen

Turtles were dissected to separate the shell, skin, and muscle tissues. The collected skin was cut into small pieces and then frozen at −20 °C for storage. The turtle skin was thawed and dried at room temperature, degreased by soaking in 0.5 M sodium carbonate (1:10 *w*/*v*) for 12 h, and subsequently soaked in 2% NaCl (1:10 *w*/*v*) for 12 h to remove non-collagenous proteins. The turtle skin was homogenized with 0.5 M glacial acetic acid (1:20 *w*/*v*). After adjusting the pH to 2, 1% pepsin was added for enzymatic digestion at 4 °C for 24 h. The solution was then centrifuged, and the supernatant was collected by filtration [31]. The precipitate was redissolved in 0.5 M acetic acid and retreated with 1% pepsin. This extraction procedure was repeated, and all supernatants were combined. After salting out (0.9 M) for 24 h, centrifugation was performed, and the collagen precipitate was dissolved in 0.5M glacial acetic acid solution, dialyzed for 24 h, and then dialyzed with deionized water for 48 h until the dialysate became chloride-free and neutral. The final product was lyophilized in a dryer (LYOQUEST-55, Azibil Telstar, Shanghai, China) and stored at −80 °C.

#### 2.2.2. Preparation of Conventional Enzymatic Collagen Hydrolysates (CE)

Sample solutions were prepared by weighing collagen dissolved in 0.02 M PBS (pH 7.5). Based on previous research [32], a collagen solution (5 mg/mL) was digested under the conditions of trypsin and alkaline protease at a compound ratio of 1:1, with 5000 U/g of enzyme added, pH 7.5, and temperatures of 50 °C for 30, 60, 90, 120, 150, and 180 min. Following centrifugation and filtration, the supernatant was collected, and the isolated collagen peptides were lyophilized and stored at −80 °C for subsequent analysis.

#### 2.2.3. Preparation of Turtle Collagen Hydrolysates by Ultrasound Pretreatment-Assisted Enzymatic Hydrolysis (UPH)

UPH was prepared according to the work of Li et al. [33], with minor modifications. The 5 mg/mL collagen solution was sonicated in an ultrasonic processor (Q700, Qsonica misonix, Newtown, CT, USA) with a probe at amplitudes of 50%, 65%, and 80% (20 kHz single frequency) for 30 min (2 s on/3 s off). Following ultrasound pretreatment, peptides were prepared using the procedure detailed in Section 2.2.2.

#### 2.2.4. Preparation of Turtle Collagen Hydrolysates by Whole-Process Ultrasound-Assisted Enzymatic Hydrolysis (WUH)

WUH was prepared by whole-process ultrasound-assisted enzymatic hydrolysis. The ultrasound was applied simultaneously with hydrolysis. The pulsed ultrasound parameter conditions are consistent with the description in Section 2.2.3 and the enzymatic conditions are consistent with Section 2.2.2.

#### 2.2.5. Determination of Degree of Hydrolysis (DH)

The DH of collagen peptides was quantified spectrophotometrically using the o-phthaldialdehyde (OPA) assay [34]. The enzymatic solution was collected from the reaction mixture at different time intervals, as described in Section 2.2.2, followed by centrifugation and filtration. A 50 μL sample solution was mixed with 3 mL of OPA reagent. After 5 min of incubation at 25 °C, absorbance was spectrophotometrically quantified at 340 nm using a UV–visible spectrophotometer (Long Jump Biotechnology Development Co. Ltd., Beijing, China). DH was calculated as follows:

(1) $$\mathrm{Serine}\;NH_{2}\;=\;\frac{OD_{\mathrm{sample}}-OD_{\mathrm{blank}}}{OD_{\mathrm{standard}}-OD_{\mathrm{blank}}}\;\times \;C_{\mathrm{Serine}-NH_{2}}\;\times \;\frac{10^{-3}\;\times \;D}{X\;\times \;P}$$

where Serine NH_2_ is the amount of free amino groups per gram of protein calculated based on serine, mmol/g; C_Serine-NH_2__ is the standard L-serine content per gram of protein with a value of 0.9516 mmol/g; D is the dilution; X is the quality of the sample, g; P is the protein content of the sample, %.

(2) $$\mathrm{DH}(\%)=\frac{{(\mathrm{Serine}\;\mathrm{NH}}_{2}-\beta )/\alpha }{H_{\mathrm{tot}}}\;\times \;100\%$$

where H_tot_ is the quantity of peptide bonds present in collagen with a value of 11.1 mmol/g; α = 0.8; β = 0.5. The values reported by Nielsen et al. [34] are used for H_tot_, α, and β.

#### 2.2.6. Determination of Molecular Weight Distribution

According to Islam et al. [13], molecular weight distribution was analyzed utilizing an HPLC system (Waters, Milford, MA, USA) equipped with a TSK gel 2000SWXL column, a 2487 UV detector, and Empower Workstation GPC software version 3.6.1. Elution used a solvent mixture of acetonitrile/water/trifluoroacetic acid (40/60/0.1, *v*/*v*/*v*) at 0.5 mL/min, with effluent monitored at 214 nm and the column temperature set to 30 °C. The MW was calculated using a calibration curve of cytochrome C (MW 12,384 Da), aprotinin (MW 6500 Da), bacitracin (MW 1422Da), Gly-Gly-Tyr-Arg (MW 451 Da), and Gly-Gly-Gly (MW 189 Da).

#### 2.2.7. Determination of Amino Acid Composition

According to Foh et al. [35], the lyophilized samples were digested with HCl (6M) at 110 °C for 24 h and then derivatized using OPA/FMOC. The amino acid composition was analyzed using an Agilent HPLC system (Agilent, Wilmington, DE, USA) with a C18 SHISEIDO column (inner diameter 4.6 nm, length 250 nm, particle size 5 μm). Eluent A comprised 0.1 M anhydrous sodium acetate/acetonitrile (97:3, *v*/*v*), with the pH adjusted to 6.5 after mixing. Chromatographic separation was performed at a column temperature of 40 °C with detection at 254 nm.

#### 2.2.8. Circular Dichroic (CD) Color Spectroscopy

We prepared a 0.1mg/mL collagen solution, following the work of Wang et al. [36]. The samples were transferred to a 0.1 cm path-length quartz cuvette for CD scanning (J-810, JASCO Corporation, Tokyo, Japan) at 10 °C under continuous nitrogen flow (3 L/min), with a wavelength range of 190–250 nm and a scanning rate of 100 nm/min.

#### 2.2.9. Scanning Electron Microscopy (SEM) Analysis

The morphological properties were observed by scanning electron microscopy (Carl Zeiss, GeminiSEM 300, Oberkochen, Germany). Lyophilized samples were mounted on a standard SEM stage with conductive adhesive, treated with ion-sprayed gold, and imaged at 100×/3000× magnifications under a 5 kV accelerating voltage.

#### 2.2.10. Surface Hydrophobicity

The surface hydrophobicity was evaluated using an 8-anilino-1-naphthalenesulfonic acid (ANS) fluorescent probe assay, with minor modifications to the protocol described by Indriani et al. [28]. Fluorescence spectra were recorded at 375 nm excitation and 400–650 nm emissions by adding 20 µL of 8 mM ANS to 5 mg/mL sample dilutions.

#### 2.2.11. Determination of Sulfhydryl and Disulfide Bond Content

A 0.5 mL sample was mixed with 2 mL Tris-glycine buffer (86 mM Tris, 90 mM Gly, pH 8). After adding 250 μL of freshly prepared DTNB solution, the mixture was incubated at 25 °C in the dark for 20 min. The absorbance was measured at 412 nm with the buffer as a blank. Sulfhydryl content was calculated as follows:

(3) $$C_{\mathrm{SH}}\left(\mu \mathrm{mol}/g\right)\;=\;\left(73.53\;\times \;A\;\times \;D\right)/C$$

where A is the absorbance value at 412 nm for different samples; C is the protein concentration—5 mg/mL; D is the dilution factor.

A 0.5 mL sample was mixed with 2 mL of Tris-glycine buffer (86 mM Tris, 90 mM Gly, 8 M urea, 0.5% SDS, pH 8), followed by 50 μL of 2-Mercaptoethanol. Absorbance was measured using the same procedure as sulfhydryl group determination, and disulfide bond content was calculated using the equation:

(4) $$C_{\mathrm{SS}}\left(\mu \mathrm{mol}/g\right)\;=\;\left((73.53\;\times \;A_{\mathrm{SS}}\;\times \;D_{S}/C_{S}\right)-C_{\mathrm{SH}})/2$$

where C_SS_ is the disulfide bond content, μmol/g; C_SH_ is the free sulfhydryl content, μmol/g; A_SS_ is the absorbance value of different samples at 412 nm; C_S_ is the concentration of the sample, 5 mg/mL; D_S_ is the dilution factor.

#### 2.2.12. Measurement of ABTS Free Radical Scavenging Rate

According to Song et al. [37], the sample (50 μL) was mixed with an ABTS^+^ working solution (3 mL) and incubated in the dark at room temperature for 6 min, followed by absorbance measurement at 734 nm. The calculation formula is as follows:

(5) $${\mathrm{ABTS}}^{+}\left(\%\right)\;=\;\frac{A_{0}-\left(A_{2}{-A}_{1}\right)\;}{A_{0}}\times \;100\%$$

where *A*_0_, *A*_1_ and *A*_2_ represent the absorbance of blank control, sample control, and sample group.

#### 2.2.13. Measurement of DPPH Free Radical Scavenging Rate

According to Huang et al. [38], 2 mL of 0.2 mM DPPH solution was combined with 2 mL sample solution. This solution was mixed thoroughly and incubated in darkness at room temperature for 30 min. The absorbance was then measured at 517 nm. The calculation formula is as follows:

(6) $$R\%\;=\;\left(1-\frac{A_{1}-A_{2}}{A_{3}}\right)\;\times \;100\%$$

where *A*_1_ is the absorbance value of the sample with DPPH solution, *A*_2_ is the absorbance value of the sample with 95% ethanol solution, and *A*_3_ is the absorbance value of the DPPH solution with 95% ethanol solution.

#### 2.2.14. Reducing Power Determination

According to Latorres et al. [39], 2 mL proteolytic solutions at concentrations of 0.1, 0.3, 0.5, 0.7, and 0.9 mg/mL were mixed with 2.5 mL of 0.2 M PBS (pH 6.6) and 2 mL of 1% (*w*/*v*) K_3_[Fe(CN)_6_]. The mixture was heated in a 50 °C water bath for 20 min, after which 2.5 mL of 10% (*w*/*v*) TCA was added. The solution was centrifuged at 5000 r/min for 10 min. Thereafter, 2.5 mL supernatant was mixed with 0.5 mL of 0.1% FeCl_3_ and 2 mL of distilled water. After reacting for 10 min, the absorbance was measured at 700 nm.

#### 2.2.15. Statistical Analysis

Each experiment was conducted in triplicate. The data were presented as mean ± standard deviation (SD). Origin 2021 and GraphPad Prism 8 were used for graphing. The statistical significance of differences was evaluated using one-way analysis of variance (ANOVA) with SPSS 26 software. Duncan’s multiple range test was performed, and *p* ≤ 0.05 was considered statistically significant.

## 3. Results

### 3.1. Effect of Different Ultrasound-Assisted Modes on the Degree of Hydrolysis

**Figure 1:**
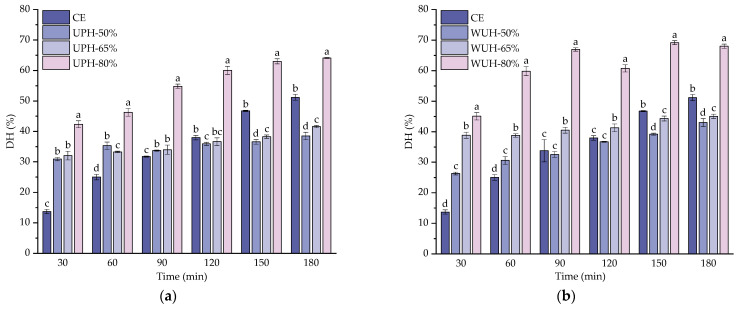
Effect of different ultrasound-assisted modes on degree of hydrolysis: (**a**) degree of hydrolysis of UPH at different ultrasonic amplitudes; (**b**) degree of hydrolysis of WUH at different ultrasonic amplitudes. Different lowercase letters indicate significant differences between different ultrasound amplitude treatment groups.

DH serves as a quantitative indicator of the protein hydrolysis rate, reflecting the number of peptide bonds cleaved during the proteolytic process [40]. The DH results for CCH under various ultrasound-assisted protease treatments are presented in Figure 1. Notably, all groups exhibited a time-dependent increase in DH, with distinct trends influenced by ultrasonic amplitude. For UPH, UPH-50% and UPH-65% showed no significant difference in DH but were both significantly lower than CE at 180 min (*p* < 0.05). In contrast, UPH-80% demonstrated significantly enhanced hydrolysis efficiency, achieving a maximum DH of 64.11 ± 0.16% at 180 min, 12.92% higher than CE (51.19 ± 0.98%, *p* < 0.05). For WUH, WUH-50% and WUH-65% showed initial DH increases followed by plateauing, with final values lower than CE at 180 min. WUH-80% exhibited continuously higher DH than CE (*p* < 0.05), peaking at 69.18 ± 0.64% at 150 min before slightly decreasing to 68.01% at 180 min. The significant DH improvement in UPH-80% and WUH-80% can be attributed to high-amplitude ultrasound-induced cavitation effects. The generated microjets likely disrupted the chemical bonds stabilizing collagen’s triple-helical structure, thereby exposing previously inaccessible peptide bonds and substantially increasing the substrate–protease interaction surface area [41]. Similar results were reported in another study, showing an increase in the DH of collagen in ultrasound-treated cowhide skins [42]. The observed decline in WUH-80% might stem from two factors. One reason is that excessive ultrasonic energy could degrade newly formed peptides into smaller inactive fragments; the second is that prolonged cavitation-induced heat might denature enzymes or promote peptide reaggregation [43]. Wang et al. [44] reported a comparable trend in research investigating the effects of ultrasound on enzymatic digestion kinetics and the bioactivity of oat protein isolate peptides.

### 3.2. Molecular Weight Distribution Analysis

The linear regression equation established for the relative molecular weight was lg(M) = −0.2427Rt + 7.005, R^2^ = 0.9931. As detailed in Table 1, the molecular weight (Mw) distributions varied with ultrasound treatment modes. All groups were dominated by peptides < 1 kDa of total peptides (CE: 90.74%, UPH: 87.69%, WUH: 89.27%), indicating the efficient enzymatic degradation of collagen macromolecules into small peptides and amino acids. Notably, the fraction of molecular weight < 0.18 kDa in UPH was higher than that of CE and WUH by 3.03% and 4.81%, whereas the molecular weight of WUH decreased by 1.78% compared to CE. The enrichment of <0.18 kDa peptides in UPH can be attributed to ultrasonic cavitation effects, including mechanical shear forces, transient high pressure, and localized heating [45]. These forces disrupt intermolecular interactions in collagen, accelerating the cleavage of peptide bonds and promoting the formation of smaller fragments. Conversely, the slight decrease of <0.18 kDa peptides in WUH may reflect prolonged ultrasonic exposure, causing peptide reaggregation via hydrophobic interactions [46].

The molecular weight distribution of collagen peptides serves as a critical determinant of their biological functionality. Extensive evidence has established that low MW hydrolysates show stronger antioxidant activity than higher Mw counterparts due to short-chain peptides’ enhanced accessibility to free radicals and improved capture efficiency [22,47]. Notably, small peptides (<3 kDa) demonstrate relatively high antioxidant potential [48], with optimal activity observed in peptides containing 5–9 amino acid residues (0.5–1.8 kDa) [49,50]. Consistent with these findings, our study revealed that approximately 25% of peptide fractions across all treatment groups were enriched within the 0.5–1 kDa range. However, a key distinction was the greater abundance of peptides in the 0.18–0.5 kDa range, likely due to the combinatorial enzymatic digestion strategy employed, as opposed to conventional single-enzyme hydrolysis. Yang et al. [51] reported that peptides in the 0.3–0.6 kDa range may correlate positively with antioxidant efficacy. These findings suggest that the most potent antioxidant peptides derived from turtle skin collagen are primarily concentrated in the 0.18–1.0 kDa molecular weight range.

### 3.3. Amino Acid Composition Analysis

The amino acid composition of CCH processed under different modalities is presented in Table 2. The predominant amino acids in the samples of CE, UPH and WUH groups were Gly (25.32%, 24.25%, 24.53%), Pro (10.52%, 10.19%, 10.27%), Ala (10.07%, 10.08%, 10.16%), and Glu (8.29%, 7.56%, 7.74%), similar to those of soft-shelled turtle collagen reported by Zou et al. [11]. Gly was the most abundant amino acid. It has been shown to contribute to intramolecular hydrogen bonding perpendicular to the helical axis, thereby stabilizing its structure [52,53]. The pyrrolidine ring of proline can also help to maintain the stability of the triple-helix structure [26].

Notably, all treatment groups exhibited substantial abundances of hydrophobic amino acids (CE: 36.80%, UPH: 38.60%, WUH: 38.17%). UPH exhibited the highest hydrophobic amino acid content with increases of 4.89% and 1.13% compared to CE and WUH, respectively. This observation aligns with previous studies indicating that hydrophobic amino acids, particularly proline (Pro), possess significant antioxidant capacity. This activity stems from the low ionization potential of Pro’s pyrrolidine ring, which facilitates its role as an effective proton/hydrogen donor [54,55]. Elevated levels of hydrophobic amino acids such as Ala, Pro, Val, Ile, and Leu may promote peptides to approach lipid radicals and enhance antioxidant properties [56]. This suggests that hydrophobicity could be a critical determinant of radical scavenging efficiency in peptides [57]. Similarly, aromatic amino acids such as Tyr and Phe, although relatively less abundant, were reported to be able to contribute to antioxidant activity through proton donation mechanisms, converting free radicals into more stable phenoxy radicals [58].

### 3.4. Circular Dichroism (CD) Spectroscopy

In general, the CD spectrum of natural triple-helix collagen is characterized by a weak positive peak at 213–230 nm and a strong negative peak at 193–204 nm [59]. The positive absorption peak at 221 nm is a circular dichroism spectroscopic signature unique to L-polyproline, with its intensity serving as a quantitative indicator of triple-helix stability. When collagen denatures and its triple-helix structure unfolds, the characteristic morphology of CD spectra, as well as the peaks, changes. Specifically, the positive absorption peak typically disappears, while the negative peak undergoes a redshift to 203–210 nm [60,61,62].

**Figure 2:**
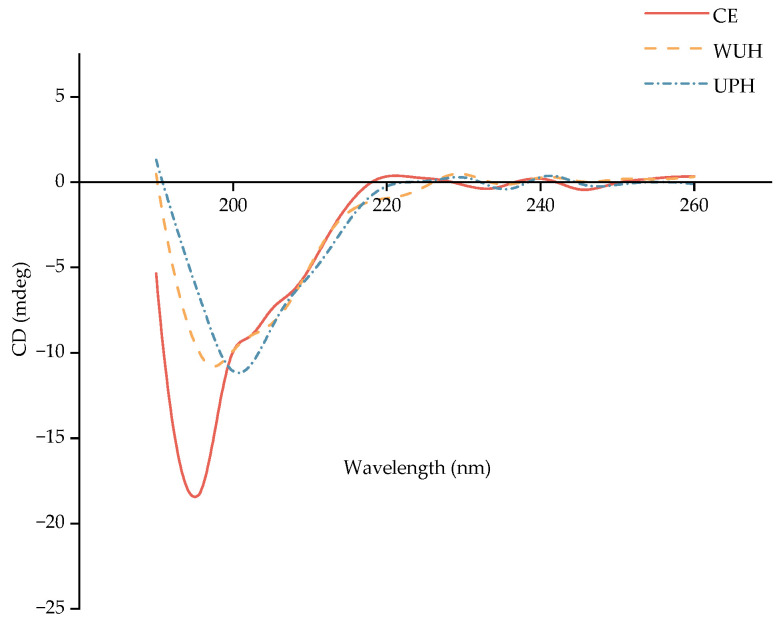
Circular dichroism of CCHS prepared in different ultrasound-assisted modes.

As shown in Figure 2, all groups (CE, UPH, WUH) lacked the 221 nm positive peak, confirming the full disruption of the triple-helix structure. Compared to CE, UPH and WUH exhibited a slight redshift of the negative peak from 196 nm to 200 nm and 198 nm, respectively, with UPH showing a more pronounced shift.

The redshift of the negative peak in UPH/WUH reflects the unfolding of the collagen backbone, as peptide chains transition from ordered helical structures to disordered conformations. UPH’s more pronounced redshift suggests stronger structural disruption, likely due to ultrasonic cavitation-induced transient high pressure and shear forces that rupture hydrogen bonds and van der Waals interactions in the collagen fibril [63,64]. This mechanism enhances enzyme accessibility to buried peptide bonds, explaining UPH’s higher yield of low-MW peptides (<1 kDa).

The alterations in the secondary structure content of CE, UPH, and WUH are illustrated in Table 3. α-helix double-negative peaks were not observed. UPH showed a 4.97% decrease in β-sheet content, a 2.6% decrease in β-turns, and a 7.56% increase in random coils compared to CE. Conversely, WUH exhibited a 9.43% increase in β-sheets, a 12.44% decrease in β-turns, and a 2.66% increase in random coils. The decrease in β-sheets observed in UPH originated from ultrasound, disrupting inter-peptide hydrogen bonds that stabilized β-sheet structures [65]. This structural perturbation exposes buried hydrophobic residues, consequently elevating surface hydrophobicity. Conversely, WUH’s increase in β-sheets may arise from the reformation of hydrogen bonds during ultrasound enzymatic treatment, favoring partial refolding into β-sheets. Meanwhile, β-sheet structures contribute to aggregate formation, which may subsequently re-bury hydrophobic amino acids [66]. The marked increase in random coils in both groups (7.56% in UPH, 2.66% in WUH) indicates collagen chain stretching and structural loosening, which correlates with the release of hydrophobic amino acids [67]. Furthermore, the increased random coil content indicates a transition from ordered secondary structures to disordered states during hydrolysis. The resulting reduction in steric constraints facilitates greater radical accessibility to reactive sites, consequently improving both scavenging efficiency and overall antioxidant activity [68]. These findings suggest ultrasound treatment may optimize antioxidant peptide production by facilitating this structural transition.

### 3.5. Scanning Electron Microscope Analysis (SEM)

**Figure 3:**
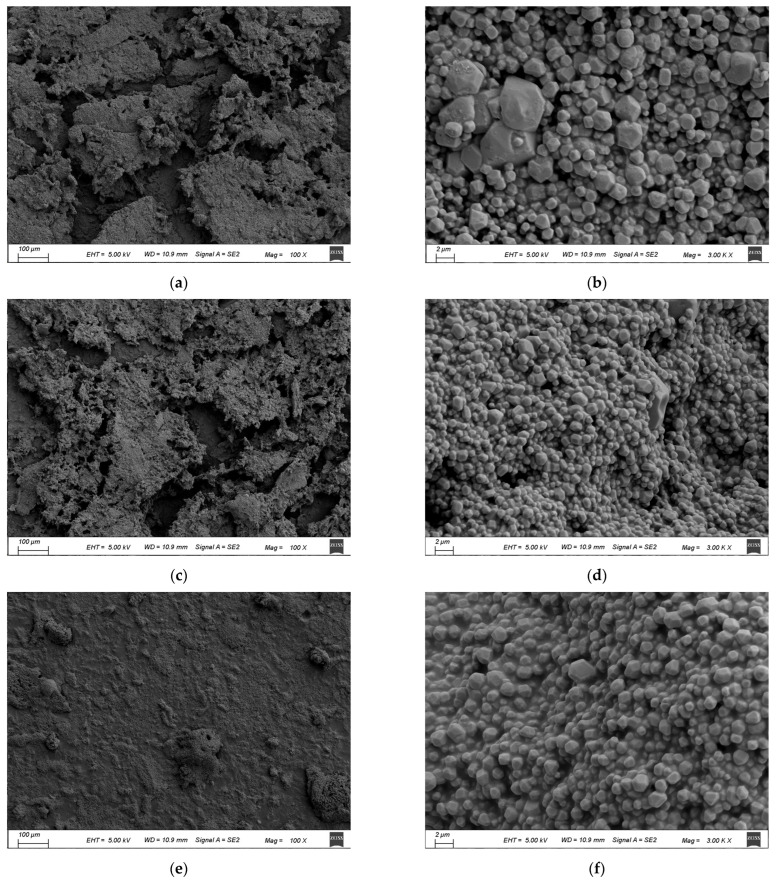
SEM images of CCHS prepared in different ultrasound-assisted modes: (**a**,**c**,**e**) represent CE, UPH, and WUH with 100× magnification; (**b**,**d**,**f**) represent CE, UPH, and WUH with 3000× magnification.

Scanning electron microscope images (Figure 3) reveal distinct surface architectures among CCH groups. The surfaces of CE, UPH and WUH were uneven and all distributed as irregular spheres and presented as granular aggregates. This may be caused by the destruction of the original collagen structure. Notably, UPH showed a looser overall morphology with smaller, more dispersed particles compared to CE, while WUH displayed a tighter distribution with occasional large aggregates. The changes in UPH may be attributed to the disruption of the collagen fiber network caused by ultrasonic cavitation, as well as the increased accessibility of enzyme action sites induced by structural changes, which results in the release of smaller peptide fragments [69]. The collagen peptide particle of WUH was also smaller compared to CE. However, there were still larger clumps of particles compared to the UPH group, which might hinder radical access to buried functional groups.

### 3.6. Surface Hydrophobicity Analysis

Surface hydrophobicity, a critical determinant of protein structure–function relationships [70], was measured using ANS fluorescence, where binding to exposed hydrophobic regions correlates with fluorescence intensity [71].

**Figure 4:**
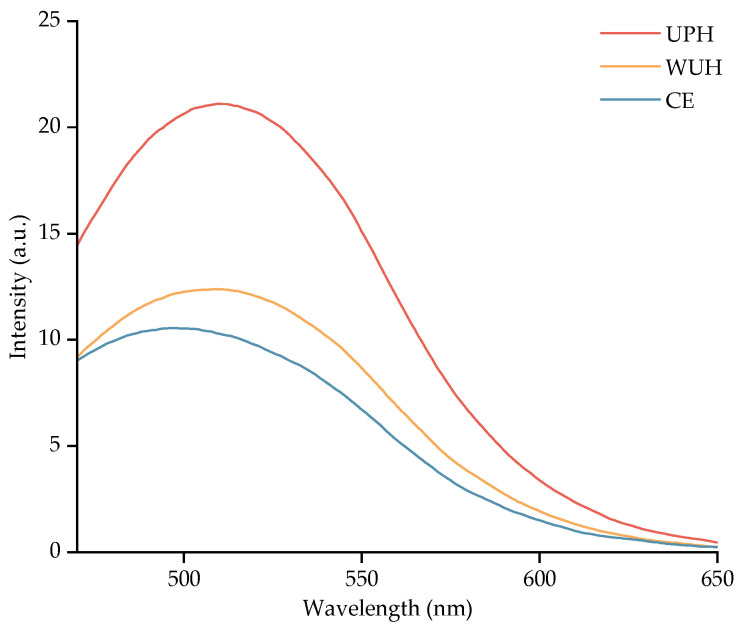
Effect of different ultrasound-assisted modes on 8 mM ANS fluorescence chromatography of CCHS.

As shown in Figure 4, both ultrasound pretreatment and whole-process ultrasound treatment can improve the surface hydrophobicity of collagen peptides. Furthermore, UPH displayed the highest degree of surface hydrophobicity. These outcomes align with previous reports by Zhou et al. [72] on the surface hydrophobicity of ultrasonically pretreated defatted wheat germ proteins. Previous studies have demonstrated that ultrasonic cavitation generates microbubbles, whose collapse produces transient high temperature, elevated pressure, shock waves, and microjets. The resulting energy and mechanical shear forces disrupt non-covalent bonds, facilitating protein molecular unfolding and enhancing the exposure of buried hydrophobic amino acid residues [46,71]. The hydrophobic groups are further released after proteolytic cleavage, thereby increasing the surface hydrophobicity of the protein. Supporting this mechanism, Singh et al. [73] demonstrated that ultrasonic treatment induces substantial tertiary structural changes in squid ovary proteins, causing molecular unfolding and enhanced exposure of hydrophobic domains during processing.

However, Kingwascharapong et al. [74] revealed that sonication initially enhances hydrophobicity in locust proteins, but prolonged exposure triggers protein polymerization or aggregation via covalent or hydrophobic interactions. This re-encapsulates exposed hydrophobic groups and reduces the surface hydrophobicity, as reported by Jia et al. [75].

### 3.7. Sulfhydryl and Disulfide Bond Content

Sulfhydryl groups represent an essential category of functional groups within proteins, playing a crucial role in the stabilization of weak secondary bonds and the maintenance of the tertiary structure of proteins [76]. The disulfide bond is an important force linking the peptide subunits in proteins, which contributes to stabilizing the spatial structure of peptide chains and maintaining protein activity in protein molecules. Both enzymatic hydrolysis and physical processing methods significantly alter the sulfhydryl–disulfide equilibrium in proteins [77].

As shown in Table 4, UP significantly increased (*p* < 0.05) free sulfhydryl content (*p* < 0.05) while concurrently reducing disulfide bond levels compared to CE. This phenomenon aligns with the work of Fu et al. [76], who reported similar ultrasound Melad-induced free sulfhydryl group exposure in glycated collagen from giant salamander skin. In contrast, US contributed to a significant decrease (*p* < 0.05) in sulfhydryl content, without the appreciable alteration of disulfide bond levels. This finding is analogous to the results of Zhou et al. [72] in their investigation of wheat germ protein isolates.

The elevated sulfhydryl content suggests that ultrasonic cavitation-induced high pressure and shear forces reduce protein particle size, exposing buried sulfhydryl groups [78]. Concurrently, the decrease in disulfide bonds indicates subunit dissociation, diminishing overall protein structural stability and hydrophobic interactions [79,80]. Compared to UPH, WUH exhibited a significant decrease (*p* < 0.05) in free sulfhydryl content concurrent with a significant increase (*p* < 0.05) in disulfide bond formation. These findings align with Wang et al.’s analysis of ultrasonicated soy proteins, where prolonged sonication similarly induced oxidative restructuring in β-conglycinin and glycinin hydrolysates [78]. Prolonged sonication generates transient radical species within cavitation bubbles, forming hydrogen peroxide. This H_2_O_2_ oxidizes free sulfhydryl groups, promoting the formation of non-native disulfide bonds that drive protein aggregation. [81,82]. Such aggregation phenomena likely sterically shield or chemically modify key antioxidant motifs in collagen peptides, ultimately reducing their measured antioxidant capacity [78].

### 3.8. In Vitro Antioxidant Activities

**Figure 5:**
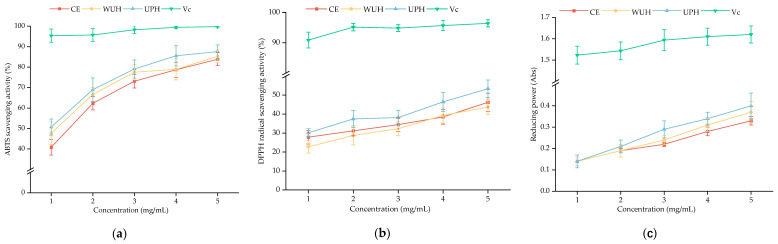
Antioxidant activity of CCHS prepared in different ultrasound-assisted modes: (**a**) ABTS radical scavenging activity of CCHS prepared in different ultrasound-assisted modes; (**b**) DPPH radical scavenging activity of CCHS prepared in different ultrasound-assisted modes; (**c**) reducing power of CCHS prepared in different ultrasound-assisted modes.

The antioxidant properties of CCH samples are summarized in Figure 5. At a peptide concentration of 5 mg/mL, UPH exhibited the highest ABTS radical scavenging rate (87.59%), outperforming the ultrasound-assisted porcine cerebral protein peptides (73%) reported by Zou et al. [83]. The half-maximal inhibitory concentrations (IC_50_) of ABTS radical scavenging were 0.99 mg/mL (UPH), 1.34 mg/mL (CE), and 1.08 mg/mL (WUH), representing 26.12% and 8.33% reductions compared to CE and WUH, respectively (*p* < 0.05). Notably, UPH exhibited superior radical scavenging capacity compared to hydrolysates from other collagen sources, with IC_50_ lower than that of colla corii asini (4.53 mg/mL), mackerel bone (2.61 mg/mL) and mackerel skin (2.50 mg/mL) [84].

For DPPH scavenging, UPH reached a maximum rate of 53.37% at 5 mg/mL, which was significantly higher than that of CE (*p* < 0.05), whereas the DPPH radical scavenging rate showed a marginal decrease in WUH. Additionally, both ultrasonication methods significantly enhanced the total reducing power of the hydrolysates (*p* < 0.05). At the 5 mg/mL concentration, UPH exhibited an absorbance of 0.404 in reducing power assays, corresponding to 21.32% and 8.02% increases relative to CE and WUH. Overall, ultrasound pretreatment (UPH) demonstrated the highest ABTS (87.59%), DPPH (53.37%), and reducing power (0.404 at 5 mg/mL). Followed by WUH, the ABTS radical scavenging rate and reducing power were higher than those of CE, although the DPPH radical scavenging rate was slightly decreased. The slight decrease in DPPH radical scavenging in WUH may be caused by the further enzymatic degradation of active peptides and loss of biological activity [44]. As previously discussed, excessive sonication creates an oxidative environment that facilitates disulfide bond formation and protein aggregation. This process subsequently obscures reactive groups and antioxidant peptides [85,86]. Furthermore, sonication-derived hydroxyl radicals can also mediate the oxidative modification of aromatic side chains—such as tyrosine and phenylalanine—resulting in the loss of their electron-donating antioxidant function [87]. Notably, ABTS scavenging activity was higher than DPPH scavenging activity in all groups at equivalent concentrations. This discrepancy likely stems from the predominance of hydrophilic amino acids in these samples, as ABTS radicals are hydrophilic whereas DPPH radicals display hydrophobic affinity [88].

The antioxidant activity of peptides is inherently linked to specific amino acid residues. Val, Tyr, Phe, Met, and His are well-recognized as key antioxidant motifs in bioactive peptides [17]. Hydrophobic amino acids, such as Ile and Met, enhance radical scavenging by donating electrons or hydrogen atoms to stabilize free radicals [89]. This is corroborated by the highest hydrophobic amino acid content in UPH in the previous section. Furthermore, ultrasound promotes the exposure of more enzymatic sites and increases the yield of low-molecular-weight peptides and free amino acids. In general, shorter-sequence peptides exhibit stronger antioxidant properties than longer-sequence peptides [90]. This is also one of the reasons why ultrasound improves the antioxidant properties of collagen peptides.

## 4. Conclusions

This study demonstrates that ultrasonic treatment can effectively alter the structure of turtle skin collagen through cavitation-mediated denaturation, enhance enzymatic hydrolysis efficiency, and generate bioactive peptides with improved antioxidant properties. Optimal ultrasonic treatment increased ABTS scavenging by 26.12% and reduced power by 21.32% compared to conventional methods, while excessive sonication duration triggered protein reaggregation and decreased bioactivity through oxidative damage mechanisms. Thus, appropriate ultrasonic conditions can facilitate the production of antioxidant peptides. This technology enables the preparation of antioxidant peptides by reutilizing waste turtle skin resources, which can serve as natural antioxidants for developing anti-aging functional foods, transdermally absorbable skincare products, or dietary supplements. Regrettably, this study did not sequence the peptides obtained from hydrolysis. Nevertheless, the observed increase in hydrophobic amino acids and low-molecular-weight components (<3 kDa) supports the formation of functional peptides. A key limitation is that the study was conducted at relatively low collagen concentrations; industrial-scale validation at higher concentrations is still required to confirm whether cavitation effects persist in viscous systems.

## Figures and Tables

**Table 1:** The molecular weight distribution of CCHS.

Molecular weight (kDa)	CE			UPH			WUH		
	Content (%)	Mn	Mw	Content (%)	Mn	Mw	Content (%)	Mn	Mw
>10	0.53	15,083	15,965	0.90	15,591	17,830	0.63	15,695	17,508
10–5	0.63	6560	6829	1.70	6597	6821	0.58	6644	6944
5–3	1.00	3697	3779	1.44	3784	3869	0.84	3755	3840
3–2	1.17	2411	2445	1.57	2401	2435	1.16	2405	2439
2–1	5.94	1276	1326	6.70	1297	1348	7.53	1265	1312
1–0.5	24.53	644	666	23.03	655	678	27.51	661	684
0.5–0.18	46.74	275	296	42.16	278	300	44.07	281	302
<0.18	19.47	/	/	22.50	/	/	17.69	/	/

Mn: number average molecular weight; Mw: weight average molecular weight.

**Table 2:** Analysis of the amino acid composition of CCHS.

Amino Acids	CE	UPH	WUH
Glycine (Gly)	253.15 ± 0.36 ^a^	242.46 ± 0.34 ^c^	245.25 ± 0.13 ^b^
Serine (Ser)	66.71 ± 0.58 ^a^	66.09 ± 0.04 ^ab^	65.90 ± 0.16 ^b^
Histidine (His)	16.27 ± 0.25 ^a^	16.32 ± 0.30 ^a^	15.99 ± 0.37 ^a^
Glutamic acid (Glu)	82.87 ± 0.85 ^a^	76.52 ± 0.51 ^b^	77.44 ± 0.42 ^b^
Aspartic acid (Asp)	67.88 ± 0.67 ^ab^	67.79 ± 0.61 ^b^	69.04 ± 0.36 ^a^
Threonine (Thr)	39.74 ± 0.53 ^a^	39.69 ± 0.48 ^a^	39.20 ± 0.04 ^a^
Arginine (Arg)	46.72 ± 0.22 ^a^	45.90 ± 0.04 ^b^	46.43 ± 0.58 ^ab^
Tyrosine (Tyr)	16.35 ± 0.36 ^b^	17.08 ± 0.09 ^a^	16.89 ± 0.29 ^ab^
Lysine (Lys)	42.29 ± 0.40 ^a^	42.12 ± 0.23 ^a^	42.14 ± 0.21 ^a^
^#^ Alanine (Ala)	100.65 ± 0.51 ^a^	100.84 ± 0.70 ^a^	101.63 ± 0.10 ^a^
^#^ Valine (Val)	49.45 ± 0.43 ^b^	50.16 ± 0.13 ^a^	48.16 ± 0.12 ^c^
^#^ Methionine (Met)	9.08 ± 0.18 ^a^	8.97 ± 0.12 ^a^	8.92 ± 0.17 ^a^
^#^ Phenylalanine (Phe)	19.40 ± 0.37 ^c^	36.90 ± 0.87 ^b^	39.00 ± 0.24 ^a^
^#^ Isoleucine (Ile)	34.17 ± 0.58 ^b^	36.27 ± 0.48 ^a^	33.38 ± 0.41 ^b^
^#^ Leucine (Leu)	50.13 ± 0.90 ^a^	51.03 ± 0.01 ^a^	47.91 ± 0.12 ^b^
^#^ Proline (Pro)	105.16 ± 0.51 ^a^	101.88 ± 0.21 ^b^	102.71 ± 0.63 ^b^
Hydrophobic amino acid	368.04 ± 0.29 ^c^	386.03 ± 1.72 ^a^	381.71 ± 0.40 ^b^

Results are expressed as residues/1000 residues; ^#^: hydrophobic amino acids; means with different letters (a–c) differ significantly (*p* < 0.05).

**Table 3:** Secondary structure content of CCHs prepared in different ultrasound-assisted modes.

Sample	α-Helix (%)	β-Sheet (%)	β-Turn (%)	Random Coil (%)
CE	-	8.97 ± 0.67 ^b^	33.47 ± 0.47 ^a^	57.57 ± 0.25 ^c^
UPH	-	4.00 ± 0.26 ^c^	30.87 ± 0.40 ^b^	65.13 ± 0.31 ^a^
WUH	-	18.40 ± 0.70 ^a^	21.03 ± 0.32 ^c^	60.23 ± 0.21 ^b^

Means with different letters (a–c) differ significantly (*p* < 0.05).

**Table 4:** Contents of sulfhydryl and disulfide bonds in CCHS prepared in different ultrasound-assisted modes.

Sample	Sulfhydryl (μmol/g)	Disulfide Bond (μmol/g)
CE	23.69 ± 0.18 ^b^	36.60 ± 0.17 ^a^
UPH	24.15 ± 0.19 ^a^	33.16 ± 0.51 ^b^
WUH	20.87 ± 0.03 ^c^	36.05 ± 0.83 ^a^

Means with different letters (a–c) differ significantly (*p* < 0.05).

## Data Availability

The original contributions presented in the study are included in the article, further inquiries can be directed to the corresponding author.

## Abbreviations

The following abbreviations are used in this manuscript:

UP	Ultrasound pretreatment
US	Ultrasound simultaneous treatment
CCHs	*Chinemys reevesii* skin collagen hydrolysates
CE	Conventional enzymatic treatment
UPH	Ultrasound pretreatment-assisted enzymatic hydrolysis
WUH	Whole-process ultrasound-assisted hydrolysates
TSE	Transmissible spongiform encephalopathy
FMD	Foot-and-mouth disease
AI	Avian influenza
ABTS	2,2-azino-bis(3-ethylbenzothiazoline-6-sulphonic acid)
DPPH	1,1-diphenyl-2-picrylhydrazyl
